# Computational Screening of Phenylamino-Phenoxy-Quinoline Derivatives against the Main Protease of SARS-CoV-2 Using Molecular Docking and the ONIOM Method

**DOI:** 10.3390/molecules27061793

**Published:** 2022-03-09

**Authors:** Suwicha Patnin, Arthit Makarasen, Pongsit Vijitphan, Apisara Baicharoen, Apinya Chaivisuthangkura, Mayuso Kuno, Supanna Techasakul

**Affiliations:** 1 Laboratory of Organic Synthesis, Department of Chemistry, Chulabhorn Research Institute, Laksi, Bangkok 10210, Thailand; suwicha@cri.or.th (S.P.); pongsit@cri.or.th (P.V.); apisara@cri.or.th (A.B.); 2 Department of Chemistry, Faculty of Science, Srinakharinwirot University, Wattana, Bangkok 10110, Thailand; apinyac@g.swu.ac.th (A.C.); mayuso@g.swu.ac.th (M.K.)

**Keywords:** molecular docking, SARS-CoV-2 main protease, coronavirus, quinoline

## Abstract

In the search for new anti-HIV-1 agents, two forms of phenylamino-phenoxy-quinoline derivatives have been synthesized, namely, 2-phenylamino-4-phenoxy-quinoline and 6-phenylamino-4-phenoxy-quinoline. In this study, the binding interactions of phenylamino-phenoxy-quinoline derivatives and six commercially available drugs (hydroxychloroquine, ritonavir, remdesivir, S-217622, N3, and PF-07321332) with severe acute respiratory syndrome coronavirus 2 (SARS-CoV-2) main protease (M^pro^) were investigated using molecular docking and the ONIOM method. The molecular docking showed the hydrogen bonding and hydrophobic interactions of all the compounds in the pocket of SARS-CoV-2 main protease (M^pro^), which plays an important role for the division and proliferation of the virus into the cell. The binding free energy values between the ligands and M^pro^ ranged from −7.06 to −10.61 kcal/mol. The molecular docking and ONIOM results suggested that 4-(2′,6′-dimethyl-4′-cyanophenoxy)-2-(4″-cyanophenyl)-aminoquinoline and 4-(4′-cyanophenoxy)-2-(4″-cyanophenyl)-aminoquinoline have low binding energy values and appropriate molecular properties; moreover, both compounds could bind to M^pro^ via hydrogen bonding and Pi-Pi stacking interactions with amino acid residues, namely, HIS41, GLU166, and GLN192. These amino acids are related to the proteolytic cleavage process of the catalytic triad mechanisms. Therefore, this study provides important information for further studies on synthetic quinoline derivatives as antiviral candidates in the treatment of SARS-CoV-2.

## 1. Introduction

Coronaviruses are enveloped, single-stranded RNA viruses that contain proteins and are covered with carbohydrates [1,2]. These pathogens can infect humans, mammals, and reptiles and may cause respiratory and digestive tract diseases in animals that can be easily spread to humans. In early 2020, a human disease caused by a new coronavirus type called severe acute respiratory syndrome coronavirus 2 (SARS-CoV-2) spread in many countries worldwide. One of the vital SARS-CoV-2 enzymes is main protease (3CL^pro^), also known as M^pro^, which is a homodimer. Each dimer is composed of three domains, designated as cysteine protease domain I–III, which contain 306 amino acids. Domains I and II are antiparallel β-barrel structures, and domain III contains five α-helices in one antiparallel globular cluster connecting to domain II [3]. The substrate binding site of M^pro^ is specific to all coronaviruses and comprises four subsites. The enzyme active site of M^pro^ contains a catalytic dyad of histidine and cysteine residues that behave as general acid/general base nucleophiles. This active site is in the cleft between domain I and II. In addition, M^pro^ cleaves at least 11 specific sites within the polyprotein during the replication process and plays an essential role in transcription to a viral gene. There is a strong conservation of residues in the binding pocket of M^pro^. Due to the essential functions of transcription and replication, M^pro^ is considered to be an important drug target for antiviral treatment [4,5,6,7].

Many medications have been used for SARS-CoV-2 treatment. However, appropriate drugs remain undiscovered; thus, indefinite drugs are being administered to patients, while suitable medications and vaccines are being explored [8,9]. Hydroxychloroquine is an antimalarial drug that inhibits heme detoxification when administered to parasitic patients. Hydroxychloroquine was selected as a treatment in the first stage of the epidemic infection of the SARS-CoV-2 virus [10]. Additionally, ritonavir, a protease inhibitor, is used as an antiretroviral drug for human immunodeficiency virus 1 (HIV-1), but with limitations due to side effects. Ritonavir is used for treating SARS-CoV-2 because of the similarities between the protease enzymes of SARS-CoV-2 and HIV-1 [11]. Recently, physicians have reported that remdesivir, a nucleoside analog drug, inhibited virus multiplication, and it has been applied against Ebola disease, MERS, and SARS [12,13]. To date, three significant drugs have been found that inhibit the function of the protease enzymes in SARS-CoV-2, namely, S-217622, N3, and PF-07321332. The oral administration of S-217622, which is a main protease inhibitor, is in a Phase III clinical trial for SARS-CoV-2 treatment. The S-217622 drug showed a preclinical pharmacokinetic profile that supported daily oral dosing as an oral therapeutic agent for treating SARS-CoV-2 [14]. N3 is also a potent inhibitor of main protease in coronaviruses, especially SARS-CoV-2. The drug N3 has exhibited potent M^pro^ inhibitor activity, with an EC_50_ value of ≥16.77 µM, for the treatment of animal infections [15,16]. However, N3 displays hepatotoxicity, which makes it carcinogenic for humans [17]. The drug PF-07321332 is an oral form developed from PF-07304814, a potent main protease inhibitor in vitro selective to human protease targets. The PF-07321332 structure consists of a nitrile warhead acting as a Michael acceptor. In addition, PF-07321332 displays potent antiviral activity against SARS-CoV-2, with an EC_90_ value of 0.181 µM [18]. PF-07321332 prevents SARS-CoV-2 replication by inhibiting primary protease, which cleaves long protein chains to the crucial parts and binds to a cysteine residue in the cysteine protease enzyme [19,20]. Nowadays, PF-07321332 is available and authorized by regulatory bodies throughout the world, such as the EMA (in the European Union) [21]. Therefore, appropriate medications against SARS-CoV-2 are under development.

In the search for novel HIV-1 non-nucleoside reverse transcriptase inhibitors (NNRTIs), phenylamino-phenoxy-quinoline derivatives have been synthesized from a combination of the pharmacophore templates of nevirapine (NVP), efavirenz (EFV), and rilpivirine (TMC278) [22,23]. These compounds can be divided into two groups, 4,6-disubstituted quinoline (**1**–**4**) and 2,4-disubstituted quinoline (**5**–**8**), and have been evaluated for their inhibitory effect on HIV-1 reverse transcriptase. A previous study indicated that 2-phenylamino-4-phenoxy-quinoline derivatives exhibited similar percentage inhibitory activity to that of NVP. The study found that those compounds might interact and bind in a similar area to NNRTIs within the pocket of HIV-1 RT. Moreover, 2-phenylamino-4-phenoxy-quinoline derivatives have shown high cytotoxic activity against some human cancer cells over normal cells and excellent binding interactions with transported proteins [24]. Because phenylamino-phenoxy-quinoline derivatives are nitrogen-containing compounds with nitrile side chains, similar to current available drugs for SARS-CoV-2, they are considered to be candidate antiviral drugs for SARS-CoV-2 treatment.

**Figure 1:**
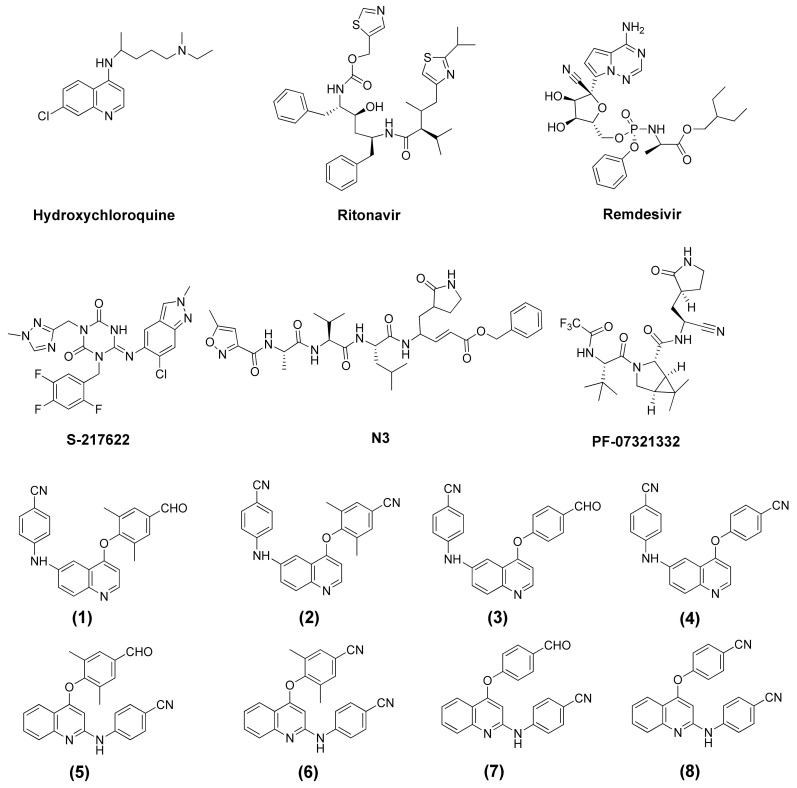
The structure of current medicaments and phenylamino-phenoxy-quinoline derivatives (**1**–**8**).

In this study, the binding interactions of presently used medications and phenylamino-phenoxy-quinoline derivatives (**1**–**8**) with SARS-CoV-2 main protease (M^pro^) were investigated using molecular docking and the ONIOM method. The structures of the compounds are shown in Figure 1. Molecular docking and the ONIOM method were used to describe the binding positions and binding interactions among biomolecules, and the results were presented as binding free energy and interaction energy values. Molecular docking and the ONIOM method have been successfully applied in most pharmaceutical research and modern drug discoveries [25,26]. Binding interactions are useful and important for understanding the function of the binding and inhibition processes. Therefore, this study has great potential for evaluating drug development for SARS-CoV-2 treatment.

## 2. Results and Discussion

### 2.1. Pharmacokinetics Study

The pharmacokinetics of the compounds were calculated using SwissADME (Developed and maintained by the Molecular Modeling Group of the SIB, Swiss Institute of Bioinformatics, Lausanne, Switzerland), which predicts the molecular properties and pharmacokinetic activity of drug-likeness. Drug-likeness is applied in screening drug candidates and is evaluated by Lipinski’s rule of five. Lipinski’s rule of five describes the absorption or permeation oral activity of a drug for the initial screening of drug-likeness and states that the molecular weight of compounds must be less than 500 Da, lipophilicity (log P) must be less than 5, H-bond donors must be less than 5, and H-bond accepters must be less than 10. Furthermore, the total polar surface area (TPSA) should be less than 140 Å^2^, and the number of rotatable bonds should be less than 10 [27,28]. According to pharmacokinetic studies of compounds, only hydroxychloroquine, PF-07321332, and compounds (**1**–**8**) correspond with Lipinski’s rule of five, together with their number of rotatable bonds being less than 10 (except PF-07321332) and TPSA less than 140 Å^2^, as shown in Table 1. Therefore, hydroxychloroquine, PF-07321332, and phenylamino-phenoxy-quinoline derivatives (**1**–**8**) are in accordance with Lipinski’s rule of five and are appropriate candidate drugs against SARS-CoV-2 main protease.

### 2.2. Molecular Docking

**Figure 2:**
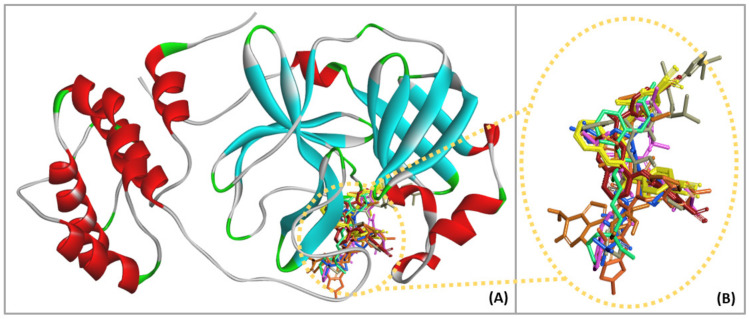
(**A**) The docking conformation of the analyzed ligands in M^pro^ using molecular docking. (**B**) Overlaying of the conformations of current medicaments: Hydroxychloroquine (green). Ritonavir (grey), remdesivir (brown), S-217622 (orange), N3 (pink), PF-07321332 (blue), and interesting compounds (**1**–**4**) (red) and (**5**–**8**) (yellow) in the binding pocket of M^pro^.

The binding interactions between the ligands in M^pro^ and the current medications, as well as the phenylamino-phenoxy-quinoline derivatives (compounds **1**–**8**), were studied using molecular docking to understand the bonding mode of the compounds in M^pro^ and to provide information for drug design. The molecular docking results, as shown in Figure 2, indicate that all selected ligands bind to the hydrophobic cavity in the binding pocket of M^pro^ (Figure 2A). Moreover, the overlaying of each selected ligand in the binding pocket reveals that all compounds are bound in a similar region (Figure 2B). The binding sites of these ligands in M^pro^ were similar to the results of previous research, in which the active compounds against SARS-CoV-2 M^pro^ interacted with the amino acid residues, namely, HIS41, ASN142, GLY143, SER144, CYS145, GLU166, ASP187, THR190, and GLN192, in the substrate binding pocket of M^pro^ via hydrogen bonding and hydrophobic interactions [18,19,20].

The binding energy values between the selected ligands and M^pro^ ranged from −7.06 to −10.61 kcal/mol, as shown in Table 2. The three lowest binding energy values of −10.61, −10.12, and −10.02 kcal/mol were observed for PF-07321332, (4-(2′,6′-dimethyl-4′-formylphenoxy)-6-(4″-cyanophenyl)-aminoquinoline (**1**), and 4-(2′,6′-dimethyl-4′-cyanophenoxy)-2-(4′cyanophenyl)-aminoquinoline (**6**), respectively. PF-07321332 had the lowest binding energy value of −10.61 kcal/mol compared with other ligands, whereas hydroxychloroquine had the highest binding energy value of −7.08 kcal/mol. However, the results showed that phenylamino-phenoxy-quinoline **1**, **6**, and **8** exhibited lower binding energy than the current medications, except for PF-07321332. Ritonavir, S-217622, and N3, a protease inhibitor, displayed binding energy values of −8.56, −9.62, and −9.44 kcal/mol with M^pro^, respectively. Remdesivir, which inhibited the RNA-dependent RNA polymerase (RdRp) of SARS-CoV-2, showed a binding energy value of −8.63 kcal/mol with M^pro^. PF-07321332 demonstrated potent in vitro antiviral activity against SARS-CoV-2, as well as activity against other coronaviruses.

**Figure 3:**
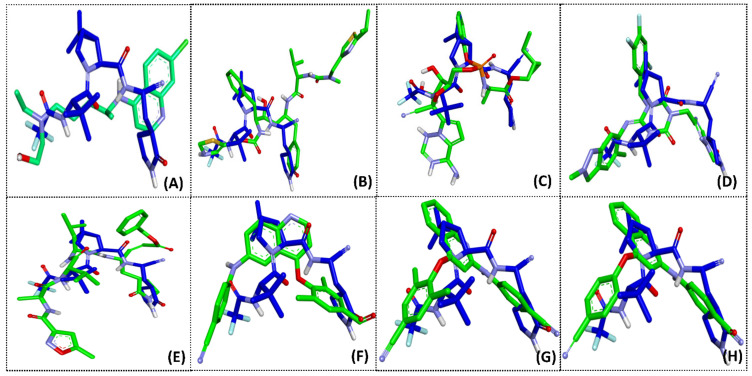
Overlay of the conformations of the analyzed ligands (green): hydroxychloroquine (**A**), ritonavir (**B**), remdesivir (**C**), S-217622 (**D**), N3 (**E**), (**1**) (**F**), (**6**) (**G**), and (**8**) (**H**) with PF-07321332 (blue) in the binding pocket of M^pro^.

Hydroxychloroquine, ritonavir, remdesivir, S-217622, N3, PF-07321332, and compounds (**1**), (**6**), and (**8**) were selected to further study the overlay of the binding interaction in the M^pro^ binding pocket and to compare the conformation of optimized ligands. The results showed that PF-07321332 had the lowest binding energy values compared with the other drugs and compounds. Then, PF-07321332 was selected to study the overlay of optimized ligands in order to consider the appropriate position in the ligand-binding sites. The optimized conformation of the compounds, namely, hydroxychloroquine, ritonavir, remdesivir, S-217622, N3, (**1**), (**6**), and (**8**) compared with PF-07321332 in the M^pro^ binding pocket are displayed in Figure 3. The results implied that S-217622, N3, (**1**), (**6**), and (**8**) were arranged in a similar position to PF-07321332 with the appropriate area in the pocket of M^pro^, except for hydroxychloroquine, ritonavir, and remdesivir (Figure 3A–C), since these compounds, which exhibited low binding energy value, contained heterocyclic skeletons with similar functional groups, such as phenyl and nitrile groups. The structure of (**1**), (**6**), and (**8**) consisted of the substituent at the 4,6- and 2,4- positions of the quinoline core structure, which differed from hydroxychloroquine in the substituents bound with M^pro^. Studies in the literature have revealed that (**1**), (**6**), and (**8**) have shown the potential to inhibit HIV-1 RT in vitro without cytotoxicity against normal cell lines [20]. Therefore, during drug development, phenylamino-phenoxy-quinoline derivatives demonstrate potential as candidates for inhibiting M^pro^ in SARS-CoV-2.

The analysis of the binding interaction between ligands and M^pro^ found that ligands can bind to the binding pocket of M^pro^ via hydrogen bonding and the Pi-Pi stacking interaction, as shown in Table 2. The formed hydrogen bonds are of two types: strong and weak. A strong hydrogen bond is formed when a hydrogen atom binds to a high electronegativity atom and is called conventional hydrogen bonding. A weak hydrogen bond is formed when a hydrogen atom binds to elements such as sulfur (S), carbon (C), or aromatic rings which serve as electron donors and, thus, are called sulfur-hydrogen bonding, carbon-hydrogen bonding, or Pi-donor-hydrogen bonding, respectively [29,30].

The molecular docking results of the binding interactions of six commercially available drugs and compounds (**1**), (**6**), and (**8**) with M^pro^ in the binding pocket surrounding a radius of 5.0 Å from M^pro^ indicated that all of these compounds bound to the hydrophobic cavity of M^pro^, as shown in Table 3. The 2D diagrams display the types of contacts between 14 compounds and M^pro^, as shown in Figures S1–S4 (see also Supplementary Materials). Phenylamino-phenoxy-quinoline (**1**), (**6**), and (**8**) bound to the amino acid residues CYS145, HIS164, GLN189, and GLN192 via conventional hydrogen bonding, to GLU166 via Pi-donor hydrogen bonding, and to HIS41 via Pi-Pi stacking interaction in the active site of M^pro^. The commercially available drugs bound to the amino acid residues HIS41, PHE140, LEU141, ASN142, GLY143, SER144, CYS145, HIS164, GLU166, LEU167, GLN189, THR190, and GLN192 via conventional hydrogen bonding, to MET49, PRO52, PHE140, MET165, and GLN189 via carbon hydrogen bonding, and to HIS41 via Pi-Pi stacking interaction in the binding pocket of M^pro^.

Studies in the literature indicate that the amino acid residues HIS41, CYS145, and ASP187 are incorporated in the catalytic triad found in the active cavity on the M^pro^ surface and are involved in proteolysis. HIS41 and CYS145 may inhibit the catalytic triad mechanism of the proteolytic cleavage process of the M^pro^, thus preventing the virus from replicating and reducing intracellular infection [31,32,33]. Moreover, the amino acid residue GLU166 plays an essential role in connecting the substrate binding site with the dimer structure of SARS-CoV-2 M^pro^ [34,35,36]. According to these results, we conclude that 4-(4′-cyanophenoxy)-2-(4″-cyanophenyl)-aminoquinoline (**8**) may have potential activity at a level similar to that of PF-07321332, because both compounds bind to M^pro^ via three amino acid residues, namely, HIS41, CYS145, and GLU166, at levels higher than other analyzed compounds.

### 2.3. ONIOM Study

**Figure 4:**
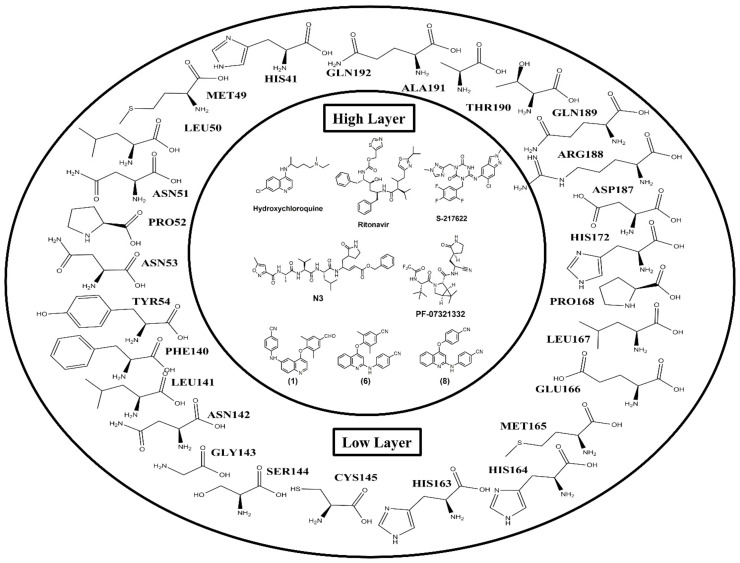
Adopted model system of ligands bound to the binding site of M^pro^.

In this study, ONIOM calculations were used to investigate the interaction mechanism of ligands with M^pro^ to obtain important information for developing new SARS-CoV-2 inhibitors. The system was divided into two layers: the high layer was treated using the B3LYP/6-31G(d,p) method, and the low layer was treated using the PM6 method (Figure 4). The interaction energy between the ligands and individual residues was calculated at the B3LYP/6-31G(d,p) level by using geometry from the X-ray structure. The interaction energy (E_int_) between each ligand and the amino acid residue was calculated by using Equation (1). ONIOM calculations were applied to confirm the results of the binding interaction between each ligand, namely, hydroxychloroquine, ritonavir, S-217622, N3, PF-07321332, **1**, **6**, and **8**, and the individual amino acid residues in M^pro^, which demonstrated each of the optimal geometries from the results of molecular docking. The small model system consisted of 26 amino acid residues within the binding pocket surrounding M^pro^ in the radius of 5.0 Å, namely, HIS41, MET49, LEU50, ASN51, PRO52, ASN53, TYR54, PHE140, LEU141, ASN142, GLY143, SER144, CYS145, HIS163, HIS164, MET165, GLU166, LEU167, PRO168, HIS172, ASP187, ARG188, GLN189, THR190, ALA191, and GLN192.

The ONIOM calculations found that all compounds bound to the substrate binding site of M^pro^ with the interaction energy values, as shown in Table 4. The three lowest total interaction energy values were found in the case of N3, PF-07321332 and S-217622, with the values of −44.73, −49.51 and −40.25 kcal/mol, respectively. These results showed that phenylamino-phenoxy-quinoline (**1**), (**6**), and (**8**) exhibited lower interaction energy than hydroxychloroquine and ritonavir. Considering phenylamino-phenoxy-quinoline derivatives, compound **1** (4,6-disubstituted quinoline) and compounds **6** and **8** (2,4-disubstituted quinoline), (**6**) and (**8**) demonstrated lower interaction energy than (**1**). Consequently, the 2,4-disubstituted quinoline derivatives interacted more with M^Pro^ than the 4,6-disubstituted quinoline derivative.

The interaction energy values between the amino acid residues, namely, HIS41, GLU166, GLN189, and GLN192 and selected ligands showed the most significant contributions because of their lowest energy. GLU166 displayed the lowest interaction energy values with the ligands compared with other amino acid residues on M^pro^, from −9.51 to −24.99 kcal/mol, indicating its important role in ligand interaction in the binding pocket of M^pro^. In addition, the crucial interactions that occurred between ligands and amino acid residues such as MET49, PHE140, GLY143, SER144, CYS145, and HIS164 presented interaction energy from −0.02 to −5.02 kcal/mol. As described in the Molecular Docking Section 2.2, the amino acids HIS41, CYS145, and GLU166 play important roles in the replication and infection of the SARS-CoV-2 virus [32,33,34]. Interestingly, (**1**), (**6**), (**8**), and PF-07321332 interacted with M^pro^ using their link with amino acid residues namely, HIS41, CYS145, HIS164, GLU166, GLN189, and GLN192. The results corresponded with the molecular docking and suggest that hydrogen bonding and the Pi-Pi stacking interaction have the most substantial contributions to the binding interaction of these amino acid residues in the binding site of M^pro^.

PF-07321332 and (**8**) showed efficient binding interactions with M^pro^; bound with three amino acid residues, HIS41, CYS145, and GLU166; and exhibited lower interaction energy than (**1**) and (**6**). Meanwhile, (**1**) and (**6**) only interacted with HIS41 and GLU166. These results suggest that the binding activity of (**8**) is similar to that of PF-07321332**.** The results demonstrate that 4-(4′-cyanophenoxy)-2-(4″-cyanophenyl)-aminoquinoline (**8**) can be exploited to identify drug candidate compounds for the development of new anti-SARS-CoV-2 main protease agents.

## 3. Materials and Methods

### 3.1. Pharmacokinetics Study

Pharmacokinetics are used to define the absorption, distribution, metabolism, and excretion (ADME) parameters which explain the various characteristics of drugs in the body. In this study, SwissADME (Developed and maintained by the Molecular Modeling Group of the SIB, Swiss Institute of Bioinformatics, Lausanne, Switzerland) was applied to analyze the pharmacokinetic parameter of ligands [37]. Drug-likeness and molecular property prediction were screened depending on Lipinski’s rule of five [25,26].

### 3.2. Ligand and Protein Structure Preparation

Six commercially available drugs (hydroxychloroquine, ritonavir, remdesivir, S217622, N3, and PF-07321332) and eight synthetic compounds of phenylamino-phenoxy-quinoline derivatives (4-(2′,6′-dimethyl-4′-formylphenoxy)-6-(4′′-cyanophenyl)-aminoquinoline (**1**), 4-(2′,6′-dimethyl-4′-cyanophenoxy)-6-4′′-cyanophenyl)-aminoquinoline (**2**), 4-(4′-formylphenoxy)-6-(4′′-cyanophenyl)-aminoquinoline (3), 4-(4′-cyanophenoxy)-6-(4′-cyanophenyl)-aminoquinoline (**4**), 4-(2′,6′-dimethyl-4′-formylphenoxy-2-(4′′-cyanophenyl)-aminoquinoline (**5**), 4-(2′,6′-dimethyl-4′-cyanophenoxy)-2-(4′′cyanophenyl)-aminoquinoline (**6**), 4-(4′-formylphenoxy)-2-(4′′-cyanophenyl)-aminoquinoline (**7**), and 4-(4′-cyanophenoxy)-2-(4′′-cyanophenyl)-aminoquinoline (**8**)) were used as ligands to study their binding interactions with the structure of M^pro^ through molecular docking and the ONIOM method. The ligands were generated using the Gaussian 16 [38] and fully optimized with the density functional theory (DFT) at the B3LYP/6-31G (d,p) level. The crystal structures of the M^pro^ enzyme in a complex with PF-07321332 were obtained from the Protein Data Bank (7VH8.pdb; RCSB Protein Data Bank, The US Research Collaboratory for Structural Bioinformatics Protein Data Bank, Rutgers, USA).

### 3.3. Molecular Docking

The ligand and water molecules in the protein structure were removed and hydrogen atoms were added. The binding interaction between the ligands and M^pro^ was simulated through the molecular docking method via AutoDock 4.2 (The Scripps Research Institute, La Jolla, CA, USA) [39], which treats the protein as a rigid structure. The Lamarckian genetic algorithm (LGA) was selected with a population size value of 150 individuals, and the number of genetic algorithms was set at 150. The grid box size was carried out at 80 Å × 80 Å × 80 Å with a spacing value of 0.375 Å. The grid center for M^pro^ was applied at the values –18.099, 17.279, and –25.630 Å. The best scoring compounds were selected and visually investigated through Accelrys Discovery Studio Client 4.0 (Accelrys, San Diego, CA, USA).

### 3.4. ONIOM Method

To analyze the enzyme–small molecule interaction, ONIOM supports accurate molecular docking and has been widely used to study antiviral activity [23,24]. The two-layered ONIOM calculations were managed using the Gaussian 16 program and optimized by DFT at the B3LYP/6-31G(d,p) level and the semi-empirical PM6 method. The interaction energy of the selected ligands (hydroxychloroquine, ritonavir, S-217622, N3, PF-07321332, (**1**), (**6**), and (**8**)) and individual amino acid residues were calculated using the B3LYP/6-31G(d,p) level of theory. The system consisted of amino acid residues surrounding the binding pocket, with the atoms of the ligands interacting within the 4 Å diameter center of the ligands, as shown in Figure 4. The residues were all assumed to be neutral amino acids and added hydrogens. The N- and C-terminal ends of cut amino acid residues were capped with acetyl group (CH3CO-) and a methyl amine group (-NHCH3), respectively. The interaction energy (E_int_) for each ligand bound to the M^pro^ was calculated using the following equation:

E_int_ = E_(residue__,ligand__)_ − E_(ligand__)_ − E_(residue__)_ (1)

where E_(residue__,ligand__)_ is the pair energy of each ligand and amino acid residue, and E_(ligand__)_ and E_(residue__)_ are the energies of ligand and each individual amino acid residue, respectively.

## 4. Conclusions

From this study, hydroxychloroquine and phenylamino-phenyloxy-quinoline derivatives (**1**–**8**) exhibited Lipinski’s rule of five and are suitable candidate drugs against SARS-CoV-2 M^pro^. The binding free energy values between the ligands and M^pro^ ranged from −7.06 to 10.61 kcal/mol; the well-known drug, PF-07321332, had the lowest binding free energy value of –10.61 kcal/mol. The binding free energy results, along with the hydrogen bonding and hydrophobic Pi-Pi stacking interactions between the selected ligands in the binding pocket of M^pro^, indicated that PF-07321332, S-217622, N3, and phenylamino-phenyloxy-quinoline derivatives (compounds **1**, **6**, and **8**) were more appropriate than that of hydroxychloroquine, ritonavir, and remdesivir. Notably, the conformations of (**1**), (**6**), and (**8**) in the M^pro^ binding pocket were arranged in a position similar to that of PF-07321332 and interacted with essential amino acid residues HIS 41, CYS145, and GLU166, which indicated their relation to the proteolytic cleavage process. Moreover, only the 4-(4′-cyanophenoxy)-2-(4′′-cyanophenyl)-aminoquinoline (**8**) case indicated hydrogen bonding interactions with CYS145 related to the catalytic triad mechanism. Thus, we concluded that 4-(4′-cyanophenoxy)-2-(4′′-cyanophenyl)-aminoquinoline (**8**) could be a new candidate for an anti-SARS-CoV-2 drug.

## Figures and Tables

**Table 1:** Molecular properties of ligands calculated by SwissADME software.

Ligand	Number of H-Bond Acceptors	Number of H-Bond Donors	LogP	Number of Rotatable Bonds	Molecular Weight (g/mol)	TPSA (Å^2^)
Hydroxychloroquine	3	2	2.35	9	335.87	48.39
Ritonavir	7	4	1.80	22	720.94	202.26
Remdesivir	12	4	0.18	14	602.58	213.36
S-217622	9	1	3.70	5	531.88	120.68
N3	9	5	0.38	22	680.79	197.83
PF-07321332	8	3	0.41	11	499.53	131.40
(1)	4	1	2.77	5	393.44	75.01
(2)	4	1	2.77	5	390.52	81.73
(3)	4	1	2.35	5	365.14	75.01
(4)	4	1	2.35	4	362.65	81.73
(5)	4	1	3.17	5	393.42	75.01
(6)	4	1	3.17	4	390.44	81.73
(7)	4	1	2.75	5	365.29	75.01
(8)	4	1	2.75	4	362.38	81.73

**Table 2:** Binding interaction between ligands with M^pro^ in 5.0 Å.

Ligand	Binding Energy (kcal/mol)	H-Bond			Pi-Pi Stacking
		a	b	c	
Hydroxychloroquine	−7.06 ± 0.11	LEU141 GLY143 CYS145 HIS164	ASN142	-	HIS41
Ritonavir	−8.56 ± 0.25	ASN142 GLY143 CYS145 GLN189	GLU166 MET165	HIS163	-
Remdesivir	−8.63 ± 0.25	GLY143 CYS145 LEU167	ASN142 GLU166	-	HIS41
S-217622	−9.62 ± 0.08	TYR54 CYS145 GLU166 GLN189	PHE140	MET165	HIS41
N3	−9.44 ± 0.16	PHE140 GLY143 GLU166	HIS41 SER144	-	-
PF-07321332	−10.61 ± 0.12	PHE140 SER144 CYS145 HIS163 HIS164 GLU166 THR190	GLN189	-	-
(1)	−10.12 ± 0.01	HIS164 GLN192	MET49 PRO52 PRO168	GLU166	HIS41
(2)	−9.75 ± 0.08	HIS164 GLN192	MET49 PRO52	GLU166	HIS41
(3)	−9.67 ± 0.04	HIS164 GLN192	MET49 PRO52 THR190	GLU166	HIS41
(4)	−9.65 ± 0.03	HIS164 GLN192	MET49 PRO52	-	HIS41
(5)	−9.72 ± 0.04	GLN189 GLN192	ASP187	GLU166	HIS41
(6)	−10.02 ± 0.01	GLN189 GLN192	MET49 ALA191	GLU166	HIS41
(7)	−9.78 ± 0.05	HIS164 GLN192	MET49 PRO52 PRO168	GLU166	HIS41
(8)	−9.97 ± 0.02	CYS145 HIS164 GLN192	MET49 PRO52	GLU166	HIS41

a: Conventional hydrogen bond. b: Carbon hydrogen bond. c: Pi-donor hydrogen bond.

**Table 3:** Distance between the ligands (current medicaments, **1**, **6** and **8**) and M^pro^ within 5.0 Å from the molecular docking.

Amino Acid Residue	Hydroxychloroquine	Ritonavir	Remdesivir	S-217622	N3	PF-07321332	(1)	(6)	(8)
HIS41	4.208	3.954	4.158	4.457	3.412	2.295	4.178	3.897	3.758
MET49	-	-	-	-	-	-	2.256	2.225	2.185
PRO52	-	-	-	-	-	-	2.694	-	2.806
PHE140	-	-	-	3.445	2.815	2.489	-	-	-
LEU141	2.015	-	-	-		-	-	-	-
ASN142	3.421	1.899	2.892	-		-	-	-	-
GLY143	2.915	2.454	2.914	-	2.203	-	-	-	-
SER144	2.036	-	-	-	-	2.725	-	-	-
CYS145	2.614	2.701	3.725	3.170	-	2.245	-	-	3.697
HIS163	-	-	-	-	-	1.877	-	-	-
HIS164	2.299	-	-	-	-	2.167	2.104	-	2.054
MET165	-	3.039	3.156	3.053	-	-	-	-	-
GLU166	-	2.964	2.937	2.943	2.089/2.263	2.012/2.192/2.954	2.962	2.432	2.937
PRO168	-	-	-	-	-	-	2.321	-	-
GLN189	-	1.984	-	2.546	-	2.352	-	3.141	-
THR190	-	-	-	-	-	2.585	-	-	-
ALA191	-	-	-	-	-	-	-	2.910	-
GLN192	-	-	-	-	-	2.838	1.954	1.872	2.251

**Table 4:** Interaction energies of ligands with the individual amino acid residues of M^pro^, calculated at the B3LYP/6-31G (d,p) level.

Amino Acid	Interaction Energy (kcal/mol)							
	Hydroxychloroquine	Ritonavir	S-217622	N3	PF-07321332	(1)	(6)	(8)
HIS41	−1.06	−0.16	−2.32	−3.42	−0.36	−2.06	−2.18	−2.2
MET49	0.64	−0.08	−0.41	−0.23	−0.87	−1.04	−1.08	−1.47
LEU50	0.06	−0.21	0.10	−0.05	0.01	−0.67	0.19	−0.43
ASN51	−0.11	−0.14	0.08	0.03	0.06	−0.42	−0.06	−0.26
PRO52	0.87	−0.14	0.74	0.01	−0.03	−0.96	−1.01	−1.86
ASN53	−0.09	0.07	0.05	0.04	0.12	−0.28	−0.07	−0.26
TYR54	−0.09	−0.27	−3.45	0.01	0.07	1.09	1.01	0.9
PHE140	1.16	−0.21	−2.32	−4.32	−3.61	0.22	−0.16	−0.47
LEU141	−2.71	−0.32	1.04	2.3	−0.77	−0.62	−0.88	0.02
ASN142	−1.36	−1.01	−1.01	−0.36	−1.21	0.46	0.77	0.21
GLY143	−3.14	−2.45	−1.25	−5.02	−0.59	0.2	0.25	0.05
SER144	0.43	−0.18	−0.24	−3.37	−3.96	−0.02	0.41	−0.07
CYS145	−2.47	−2.4	−2.89	−2.5	−3.44	−0.67	−0.98	−2.87
HIS163	−0.78	−1.69	−1.14	−2.08	−3.05	−0.57	−0.49	−0.66
HIS164	−3.2	−1.07	−1.33	0.15	−2.75	−2.98	−0.55	−3.16
MET165	2.29	0.94	−1.89	0.03	0.13	0.48	1.53	1.5
GLU166	−9.51	−18.44	−22.49	−23.14	−24.99	−14.47	−21.33	−21.75
LEU167	−0.94	−0.82	−0.22	−0.64	−0.32	0.09	0.17	0.11
PRO168	−0.42	0.25	0.10	−0.08	−0.05	−1.09	−0.03	−0.83
HIS172	−0.19	−0.19	−0.67	−2.28	−0.21	0.26	−0.18	−0.76
ASP187	2.57	−0.26	0.22	0.49	−1.03	−0.44	−0.43	0.02
ARG188	−3.2	0.33	0.98	−0.45	0.49	−0.31	−1.17	−0.57
GLN189	−0.44	−3.03	−3.17	−0.08	−3.54	−1.36	−2.15	−1.07
THR190	−0.22	1.48	1.01	0.07	0.21	−0.85	−0.31	0.42
ALA191	−0.18	−0.06	0.02	0.09	0.05	−0.36	−1.19	−0.78
GLN192	−0.88	−0.41	0.21	0.07	0.13	−5.31	−3.38	−3.66
Total	−22.97	−30.47	−40.25	−44.73	−49.51	−31.68	−33.30	−39.90

## Data Availability

Not applicable.

## Supplementary Materials

The following supporting information can be downloaded at: https://www.mdpi.com/article/10.3390/molecules27061793/s1, Figures S1–S4: 2D diagram showing the types of contacts formed between ligands and M^pro^.

## Sample Availability

All synthetic compounds are available.
