# Supplementary material for: Computational Screening of Phenylamino-Phenoxy-Quinoline Derivatives against the Main Protease of SARS-CoV-2 Using Molecular Docking and the ONIOM Method
Source: Molecules. 2022 Mar 9;27(6):1793. doi: 10.3390/molecules27061793 (PMC8955101; doi:10.3390/molecules27061793)
Supplement: Supplementary file 1 [file molecules-27-01793-s001.zip › molecules-1620550-supplementary.pdf]

molecules

MDPI

Supplementary Data

Computational Screening of Phenylamino-Phenoxy-Quinoline Derivatives Against the Main Protease of SARS-CoV-2 Using Molecular Docking and the ONIOM Method

Suwicha Patnin[1], Arthit Makarasen[1,*], Pongsit Vijitphan[1], Apisara Baicharoen[1], Apinya Chaivisuthangkura[2], May-uso Kuno[2], Supanna Techasakul[1,*]

1 Department of Chemistry, Laboratory of Organic Synthesis, Chulabhorn Research Institute, Laksi, Bangkok 10210, Thailand; suwicha@cri.or.th (S.P.); pongsit@cri.or.th (P.V.); apisara@cri.or.th (A.B.)

2 Department of Chemistry, Faculty of Science, Srinakharinwirot University, Wattana, Bangkok 10110, Thailand; apinyac@g.swu.ac.th (A.C.); mayuso@g.swu.ac.th (M.K.)

* Correspondence: arthit@cri.or.th (A.M.); supanna@cri.or.th (S.T.)

The molecular docking results of the binding interactions of hydroxychloroquine, ritonavir, and remdesivir with M^{pro} in the binding pocket surrounding within the radius of 5.0 Å from M^{pro} as described below.

The amino acid residues of M^{pro} surrounding hydroxychloroquine within 5.0 Å were HIS41, MET49, TYR54, PHE140, LEU141, GLY143, SER144, CYS145, HIS163, HIS164 MET165, GLU166, HIS172 and ARG188. The conventional hydrogen bonding interactions between M^{pro} and hydroxychloroquine were demonstrated as between the O atom of the C=O group in LEU141 and the H atom of OH group in hydroxychloroquine, between the N atom of the NH2 group in GLY143 and the O atom of OH group in hydroxychloroquine, between the N atom of the NH2 group in CYS145 and the H atom of OH group in hydroxychloroquine, and between the O-atom of the C=O group in HIS164 and the H-atom of NH2 group in hydroxychloroquine. In addition, one of the carbon hydrogen bond interacted with ASN142 and Pi-Pi stacking interaction between HIS41 residue of M^{pro} and benzene ring of hydroxychloroquine was observed according to Figure S1.

In the case of ritonavir, the amino acid residues of M^{pro} surrounding ritonavir within 5.0 Å were HIS41, MET49, TYR54, PHE140, LEU141, ASN142, GLY143, SER144, CYS145, HIS163, HIS164, MET165, GLU166, LEU167, PRO168, ASP187, ARG188 and GLN189. The conventional hydrogen bonding interactions between M^{pro} and ritonavir were shown as between the H atom of the SH group in CYS145 and the N atom of NH2 group in ritonavir, between the O-atom of the C=O group in ASN142 and the H-atom of NH2 group in ritonavir, between the O atom of the C=O group in GLN189 and the N atom of NH2 group in ritonavir, and between the H atom of the NH2 group in GLY143 and the O atom of C=O group in ritonavir. One of the carbon hydrogen bond formed with MET165 and one of Pi-Donor displayed with GLU166. Moreover, HIS163 of M^{pro} interacted with ritonavir by Pi-donor hydrogen bond as shown in Figure S1.

Figure S1 demonstrated that the amino acids surrounding remdesivir within 5.0 Å were HIS41, MET49, PHE140, LEU141, ASN142, GLY143, SER144, HIS163, MET165, GLU166, LEU167, PRO168, HIS170, ARG188, GLN189, THR190, ALA191 and GLN192. Five conventional hydrogen bonding interactions were formed as between the H atom of the SH group in CYS145 and the N atom of NH2 group in remdesivir, between the O-atom of the C=O group in LEU167 and the H-atom of OH group in remdesivir, between the O

atom of the C=O group in GLU166 and the N atom of OH group in remdesivir, and between the H atom of the NH2 group in GLY143 and the O atom of OH group in remdesivir. Two of the carbon hydrogen bonds with ASN142 and GLU166 were observed. HIS41 residue bound to benzene ring of remdesivir via Pi-Pi stacking interaction.

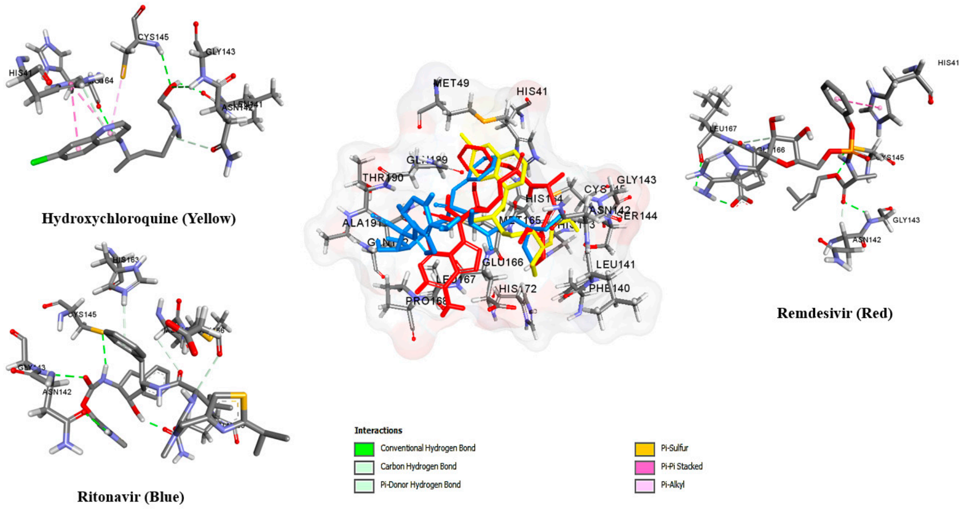

Figure S1. 2D diagram showing the types of contacts formed between hydroxychloroquine, ritonavir, remdesivir and M^{pro}.

The molecular docking results of the binding interactions of S-217622, N3, and PF-07321332 with M^{pro} in the binding pocket surrounding within the radius of 5.0 Å from M^{pro} as described below.

The amino acid residues of M^{pro} surrounding S-217622 within 5.0 Å were HIS41, MET49, TYR54, PHE140, LEU141, GLY143, SER144, CYS145, HIS163, HIS164 MET165, GLU166, HIS172 and ARG188. The conventional hydrogen bonding interactions between M^{pro} and S-217622 were demonstrated as between the H atom of NH2 group in TYR54 and the F atom of benzyl group in S-217622, between the H atom of the SH group in CYS145 and the N atom of NH2 group in S-217622, between the O atom of the C=O group in GLU166 and the H atom of NH2 group in S-217622, and between the H atom of the NH2 group in GLN192 and the F atom of benzyl group in S-217622. One of the carbon hydrogen bond interacted with PHE140 and One of the Pi-donor hydrogen bond formed with MET165. Pi-Pi stacking interaction between HIS41 residue of M^{pro} and benzene ring of S-217622 was observed according to Figure S2.

For the N3, the amino acid residues of M^{pro} surrounding N3 within 5.0 Å were HIS41, MET49, PRO52, TYR54, CYS145, HIS163, HIS164, MET165, GLU166, LEU167, PRO168, ASP187, ARG188, GLN189, THR190, ALA191 and GLN192. The conventional hydrogen bonding interactions between M^{pro} and N3 were demonstrated as between the O atom of the C=O group in PHE140 and the H atom of NH2 group in N3, and between the H atom of the NH2 group in GLY143 and the O atom of the C=O group in N3. In addition, two conventional hydrogen bonding interactions were formed as between the O atom of the C=O group in GLU166 and the H atom of NH2 group in N3, and between the O atom of the C=O group in GLU166 and the H atom of NH2 group in N3. Two of the carbon hydrogen bonds interacted with HIS41 and SER144.

In the case of PF-07321332, PF-07321332 bound to the hydrophobic cavity in the binding pocket of M^{pro} as shown in Figure S2. The results indicated that the amino acid residues of M^{pro} surrounding PF-07321332 within 5.0 Å were HIS41, MET49, PRO52, TYR54, HIS163, HIS164, MET165, GLU166, PRO168, ARG188, THR190, ALA191 and GLN192. The conventional hydrogen bonding interactions between M^{pro} and PF-07321332 were demonstrated as between the H atom of the NH2 group in HIS41 and the N atom of CN group in PF-07321332, between the N atom of the NH2 group in PHE140 and the H atom of NH2 group in PF-07321332, between the H atom of the OH group in SER144 and the O atom of C=O group in PF-07321332, between the H atom of the SH group in CYS145 and the N atom of NH2 group in PF-07321332, between the H atom of the NH2 group in HIS163 and the O atom of C=O group in PF-07321332, between the O atom of the C=O group in HIS164 and the H atom of NH2 group in PF-07321332, between the N atom of the NH2 group in THR190 and the O atom of C=O group in PF-07321332, and between the N atom of the NH2 group in GLN192 and the O atom of C=O group in PF-07321332. Furthermore, three conventional hydrogen bonding interactions were formed as between the O atom of the C=O group in GLU166 and the H atom of NH2 group in PF-07321332, between the O atom of the C=O group in GLU166 and the H atom of NH2 group in PF-07321332, and between the N atom of the NH2 group in GLU166 and the O atom of C=O group in PF-07321332. Two of the carbon hydrogen bonds displayed with LEU167 and GLN189.

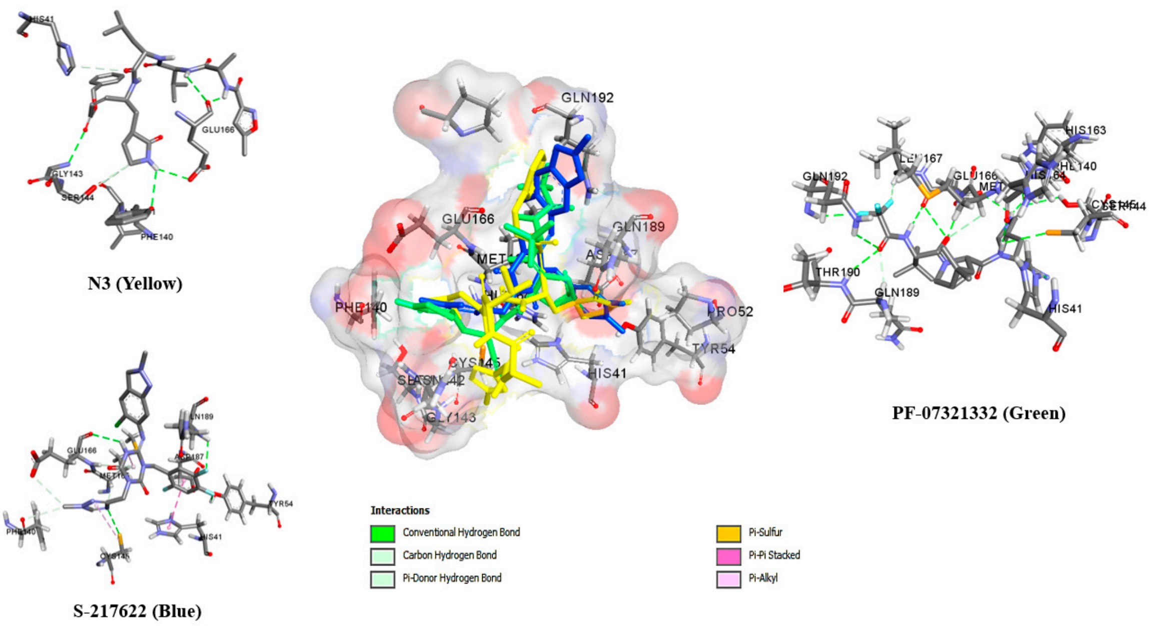

Figure S2. 2D diagram showing the types of contacts formed between S217622, N3, PF-07321332, and M^{pro}.

The molecular docking results of the binding interactions of ligands (**1-4**) with M^{pro} in the binding pocket surrounding within the radius of 5.0 Å from M^{pro} as described below

For the 6-phenylamino-4-phenyloxy-quinoline (**1**), the amino acid residues of M^{pro} surrounding (**1**) within 5.0 Å were HIS41, MET49, PRO52, TYR54, CYS145, HIS164, MET165, GLU166, LEU167, PRO168, ASP187, ARG188, GLN189 THR190 and GLN192. The conventional hydrogen bonding interactions between M^{pro} and (**1**) were demonstrated as between the H atom of the NH2 group in GLN192 and the O atom of C=O group in (**1**), and between the H atom of the NH2 group in HIS164 and the N atom of NH2 group in (**1**). Three of the carbon hydrogen bonds formed with MET49, PRO52 and PRO168, one of the Pi-Donor hydrogen bond displayed with GLU166. Moreover, one hydrophobic interaction was performed using Pi-Pi stacking between M^{pro} and (**1**). This interaction was shown as between HIS41 residues and the benzene ring of (**1**) (Figure S3).

In the case of 4-(2′,6′-Dimethyl-4′-cyanophenoxy)-6-(4′′-cyanophenyl)-aminoquinoline (**2**), (**2**) bound to the hydrophobic cavity in the binding pocket of M^{pro} as shown in Figure S3. The results indicated that the amino acid residues of M^{pro} surrounding (**2**) within 5.0 Å were HIS41, MET49, PRO52, TYR54, HIS163, HIS164, MET165, GLU166, PRO168, ARG188, THR190, ALA191 and GLN192. The conventional hydrogen bonding interactions between M^{pro} and (**2**) were demonstrated as between the H atom of the NH2 group in GLN192 and the N atom of CN group in (**2**), and between the O atom of the C=O group in HIS164 and the H atom of NH2 group in (**2**). Two of the carbon hydrogen bonds formed with MET49 and PRO52, one of the Pi-Donor hydrogen bond showed with GLU166. Besides, one hydrophobic interaction was performed using Pi-Pi stacking between HIS41 and (**2**).

Figure S3 demonstrated that 4-(4′-formylphenoxy)-6-(4′′-cyanophenyl)-aminoquinoline (**3**) bound to the hydrophobic cavity in the binding pocket of M^{pro} within the radius of 5.0 Å, surrounded by HIS41, MET49, PRO52, TYR54, CYS145, HIS163, HIS164, MET165, GLU166, LEU167, PRO168, ASP187, ARG188, GLN189, THR190, ALA191 and GLN192. Two conventional hydrogen bonding interactions were formed between M^{pro} and (**3**) which exhibited between the H atom of the NH2 group in GLN192 and the N atom of CN group in (**3**), and between the O atom of the C=O group in HIS164 and the H atom of NH2 group in (**3**). Three of the carbon hydrogen bonds interacted with MET49, PRO52, and THR190. One of the Pi-Donor hydrogen bond showed with GLU166. One hydrophobic interaction was also observed between SARS-CoV-2 and (**3**) using Pi-Pi stacking interaction between HIS41 residue and benzene ring of (**3**).

For the 4-(4′-cyanophenoxy)-6-(4′′-cyanophenyl)-aminoquinoline (**4**), the amino acid residues of M^{pro} surrounding (**4**) within 5.0 Å were HIS41, MET49, PRO52, TYR54, CYS145, HIS163, HIS164, MET165, GLU166, LEU167, PRO168, ASP187, ARG188, GLN189, THR190, ALA191 and GLN192. The conventional hydrogen bonding interactions between M^{pro} and (**4**) were demonstrated as between the H atom of the NH2 group in GLN192 and the O atom of C=O group in (**4**), and between the H atom of the NH2 group in HIS164 and the N atom of NH2 group in (**4**). Two of the carbon hydrogen bonds with MET49 and PRO52 were depicted in Figure S3. One hydrophobic interaction was also observed between SARS-CoV-2 and (**4**) using the Pi-Pi stacking interaction between HIS41 residue and benzene ring of (**4**).

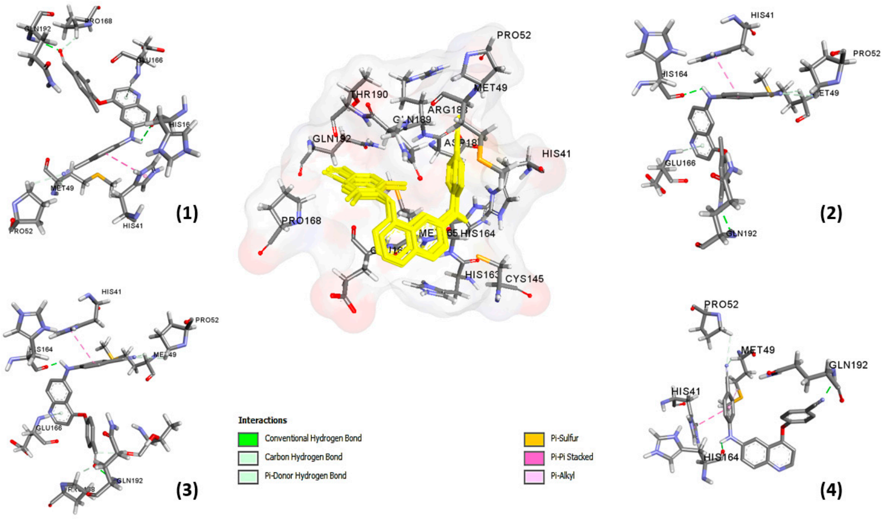

Figure S3. 2D diagram showing the types of contacts formed between 4,6-disubstituted quinoline (**1**-**4**) and M^{pro}.

The molecular docking results of the binding interactions of ligands (**5-8**) with M^{pro} in the binding pocket surrounding within the radius of 5.0 Å from M^{pro} as described below.

For the 2-phenylamino-4-phenyloxy-quinoline (**5**), the amino acid residues of M^{pro} surrounding (**5**) within 5.0 Å were HIS41, MET49, PRO52, TYR54, CYS145, HIS163, HIS164, MET165, GLU166, LEU167, PRO168, ASP187, ARG188, GLN189, THR190, ALA191 and GLN192. The conventional hydrogen bonding interactions between M^{pro} and (**5**) were demonstrated as between the O atom of the C=O group in HIS164 and the H atom of NH2 group in (**5**)**,** and between the H atom of the NH2 group in GLN192 and the N atom of CN group in (**5**). One of the carbon hydrogen bond showed with ASP187 and one of the Pi-Donor hydrogen bond displayed with GLU166. Pi-Pi stacking interaction between HIS41 residue and benzene ring of (**5**) was depicted in Figure S4.

In the case of 4-(2′,6′-dimethyl-4′-cyanophenoxy)-2-(4′′ cyanophenyl)-aminoquinoline (**6**), (**6**) bound to the hydrophobic cavity in the binding pocket of M^{pro} as shown in Figure S4. The results indicated that the amino acid residues of M^{pro} surrounding (**6**) within 5.0 Å were HIS41, MET49, PRO52, TYR54, HIS163, HIS164, MET165, GLU166, PRO168, ARG188, THR190, ALA191 and GLN192. The conventional hydrogen bonding interactions between M^{pro} and (**6**) were demonstrated as between the H atom of the NH2 group in GLN189 and the N atom of CN group in (**6**), and between the H atom of the NH2 group in GLN192 and the N atom of CN group in (**6**). Two of the carbon hydrogen bonds formed with MET49 and ALA191. One of the Pi-Donor hydrogen bond showed with GLU166 and one of Pi-sulfur displayed with CYS145. One of Pi-Pi stacking interaction between HIS41 residue and the benzene ring of (**6**) was observed.

The amino acid residues of M^{pro} surrounding 4-(4′-formylphenoxy)-2-(4′′-cyanophenyl)-aminoquinoline (**7**) within 5.0 Å were HIS41, MET49, PRO52, TYR54, CYS145, HIS163, HIS164, MET165, GLU166, LEU167, PRO168, ASP187, ARG188, GLN189, THR190, ALA191 and GLN192. The conventional hydrogen bonding interactions between M^{pro} and (**7**) were demonstrated as between the H atom of the NH2 group in GLN192 and the O atom of C=O group in (**7**), and between the H atom of the NH2 group in HIS164 and the N atom of NH2 group in (**7**). Three of the carbon hydrogen bonds with MET49, PRO52 and PRO168 were observed. One of the Pi-Donor hydrogen bond displayed with GLU166. Moreover, one hydrophobic interaction was performed using Pi-Pi stacking between M^{pro} and (**7**). This interaction was shown as between HIS41 residues and the benzene ring of (**7**).

Figure S4 demonstrated that 4-(4′-cyanophenoxy)-2-(4′′-cyanophenyl)-aminoquinoline (**8**) bound to the hydrophobic cavity in the binding pocket of M^{pro} within the radius of 5.0 Å, surrounded by HIS41, MET49, PRO52, TYR54, CYS145, HIS163, HIS164, MET165, GLU166, LEU167, PRO168, ASP187, ARG188, GLN189, THR190, ALA191 and GLN192. Three conventional hydrogen bonding interactions were formed between M^{pro} and (**8**) which exhibited between the H atom of the SH group in CYS145 and the N atom of NH2 group in (**8**), between the O atom of the C=O group in HIS164 and the H atom of NH2 group in (**8**), and between the H atom of the NH2 group in GLN192 and the N atom of CN group in (**8**). Two of the carbon hydrogen bonds showed with MET49 and PRO52. One of the Pi-Donor hydrogen bond with GLU166. One hydrophobic interaction was also demonstrated between SARS-CoV-2 and (**8**) using Pi-Pi stacking interaction between HIS41 residue and benzene ring of (**8**).

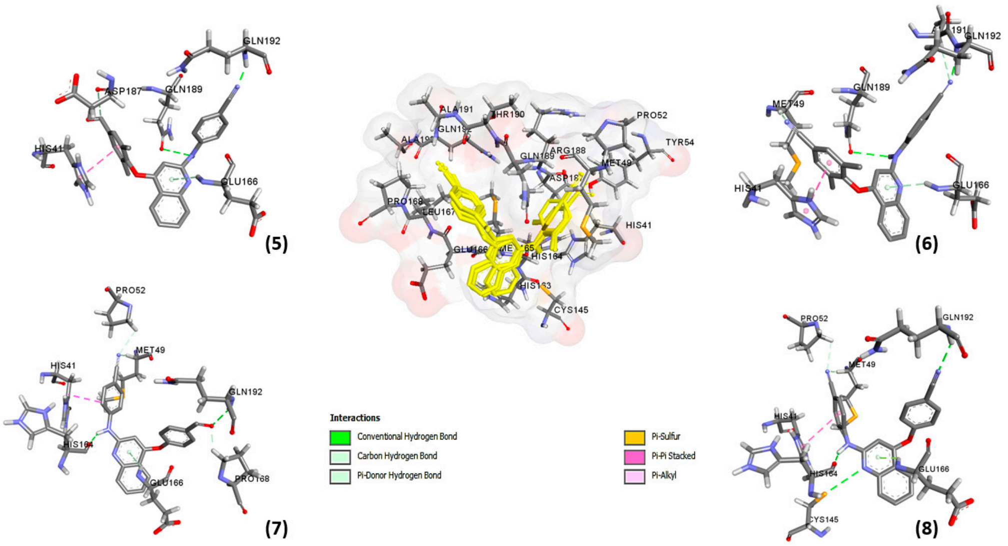

Figure S4. 2D diagram showing the types of contacts formed between 2,4-disubstituted quinoline (**5**-**8**) and M[Pro].
